# Optimization of 3-D organotypic primary colonic cultures for organ-on-chip applications

**DOI:** 10.1186/1754-1611-8-9

**Published:** 2014-04-01

**Authors:** Asad A Ahmad, Yuli Wang, Adam D Gracz, Christopher E Sims, Scott T Magness, Nancy L Allbritton

**Affiliations:** 1 Department of Biomedical Engineering, University of North Carolina, Chapel Hill, NC, 27599 and North Carolina State University, Raleigh, NC 27695, USA; 2 Department of Chemistry, University of North Carolina, Chapel Hill, NC 27599, USA; 3 Department of Medicine, Division of Gastroenterology and Hepatology, University of North Carolina, Chapel Hill, NC 27599, USA; 4 Department of Cell Biology and Physiology, University of North Carolina, Chapel Hill, NC 27599, USA

## Abstract

**Background:**

New advances enable long-term organotypic culture of colonic epithelial stem cells that develop into structures known as colonoids. Colonoids represent a primary tissue source acting as a potential starting material for development of an *in vitro* model of the colon. Key features of colonic crypt isolation and subsequent colonoid culture have not been systematically optimized compromising efficiency and reproducibility. Here murine crypt isolation yield and quality are optimized, and colonoid culture efficiency measured in microfabricated culture devices.

**Results:**

An optimal incubation time of 60 min in a chelating buffer released 280,000 ± 28,000 crypts from the stroma of a single colon with 79.3% remaining intact. Mechanical agitation using an average acceleration of 1.5 × g liberated the highest quality crypts with 86% possessing well-defined lumens. Culture in 50% Matrigel resulted in the highest colonoid formation efficiency of 33 ± 5%. Immunostaining demonstrated that colonoids isolated under these conditions possessed stem/progenitor cells and differentiated cell lineages. Microfabrication substrates (glass, polystyrene, PDMS, and epoxy photoresists: SU-8 and 1002-F) were tested for compatibility with colonoid culture. PDMS promoted formation of 3-D colonoids containing stem/progenitor cells, while other substrates promoted outgrowth of a 2-D epithelial monolayer composed of differentiated cells.

**Conclusion:**

Improved crypt isolation and 3-D colonoid culture, along with an understanding of colonic epithelial cell behavior in the presence of microfabrication substrates will support development of ‘organ-on-a-chip’ approaches for studies using primary colonic epithelium.

## Background

Self-renewal of the colonic epithelium is driven by the proliferation of epithelial stem cells located at the base of the functional tissue subunit called the colon crypt. The rapid regeneration to renew the epithelium is driven by colonic epithelial stem cells (CESCs). Understanding the CESC biology and conditions impacting their growth and differentiation is an active area of research [1-3]. The colonic epithelium is negatively impacted by a number of inflammatory diseases, cancer and acute injuries. The high incidence of colorectal cancer (CRC) in the Western World is believed to be in part due to the high proliferation rate of the epithelial lining, and increasing evidence strongly suggests CRC may arise at the level of the stem cell [4,5]. Inflammatory bowel diseases including ulcerative colitis and Crohn’s disease result from attack on the crypt cells by inflammatory infiltrates [6,7]. Due to technical challenges for the *in vitro* assessment of colonic mucosa and crypts, studies of colonic physiology and pathophysiology have been restricted primarily to *in vivo* inspection. *In vivo* studies by endoscopy or noninvasive imaging have enabled examination of living colonic tissue at a macroscopic, but not cellular scale. Histological evaluation of fixed tissue has permitted study at the cellular level, but with the loss of the rich and dynamic qualities of the living tissue.

Recent breakthroughs in the understanding of fundamental morphogenetic pathways and their contributions to intestinal homeostasis have enabled culture methods to be devised that successfully generate 3-D crypt-like cellular spheroids, or ‘colonoids’, from isolated crypts or purified stem cells [3,8-11]. Colonoids are sustainable *in vitro* for long periods up to a year. Rapid *ex vivo* establishment of colonoids in culture is potentiated by three factors: Wnt-3A, the Wnt-agonist R-spondin1 and the BMP-antagonist Noggin. *In vivo*, Wnt-3A is essential for stem cell maintenance [8]; excess R-spondin1 induces hyperplasia; Noggin at supraphysiologic concentrations produces an expansion in crypt numbers [11]. In the colonoid-culture system, CESCs, progenitors and differentiated cell lineages are present, and their composition can be adjusted by the concentration of these and other growth factors [3]. The availability of the colonoid culture system is expected to open the door to future investigations into the CESC niche and the contribution of morphogenetic cues in crypt homeostasis and organization [12]. These organotypic culture methods will have widespread impact on studies of intestinal biology, host-pathogen interactions, neoplasia and regenerative medicine. Furthermore, a better understanding of the optimal crypt isolation and culture conditions may enable the creation of novel microscale devices to recapitulate gut function *in vitro* using primary cells.

While three-dimensional (3-D) cell culture systems better mimic the microstructure of intact organs relative to 2-D cultures, the 3-D systems still fail to fully recapitulate organ-level physiologic functions presumably due to an inability to fully control the microenvironment of the organoid. Consequently, a growing trend is to build ‘organ-on-chip’ devices which integrate the 3-D tissue culture systems with microdevice technologies to offer enhanced control of both surface and fluidic conditions [13-16]. However due to the difficulty in obtaining and isolating primary tissue, these ‘organ-on-chip’ devices often utilize tumor cell lines which are incapable of demonstrating organ-level physiologic function. For example, ‘gut-on-chips’ devices are frequently assembled by placing Caco-2 tumor cells within microdevices [17-19]. The Caco-2 tumor cell line has been adapted for tissue culture and poorly mimics the intestinal epithelium in terms of architecture, growth factor response, differentiation, gene expression and susceptibility to apoptosis [20,21]. A significant challenge to the ‘organ-on-chip’ community is the development of optimized strategies to isolate high-quality primary cells for culture within a microdevice.

Although recent work has enhanced *in vitro* intestinal culture, isolation of the crypts and propagation of the colonoids has not been systematically optimized. Existing protocols fail to quantify overall yield and viability of the isolated crypts over time. The current work focuses on maximizing the yield of viable, high-quality crypts obtained from resected colon and enhancing the overall culture efficiency to produce large numbers of living colonoids from the isolated crypts. Since the cell microenvironment impacts colon cell fate and function, further characterization of the matrix concentration and identification of biocompatible substrates for colonoid culture were also performed. Microengineered environments are increasingly used to direct tissue and stem cell organization so that commonly used materials for microfabrication (including glass, polydimethoxysilane, polystyrene and epoxy photoresists), were assessed for their ability to support colonoid formation. This paper focuses on three major points of emphasis: 1) standardization of crypt isolation protocol, 2) optimization of Matrigel concentration for colonoid formation, and 3) crypt cell interaction with various substrates. We believe this research will support future development of intestinal studies and ‘organ-on-chip’ endeavors.

## Results and discussion

### Optimization of incubation time with chelating agents to remove epithelium from basement membrane

**Figure 1:**
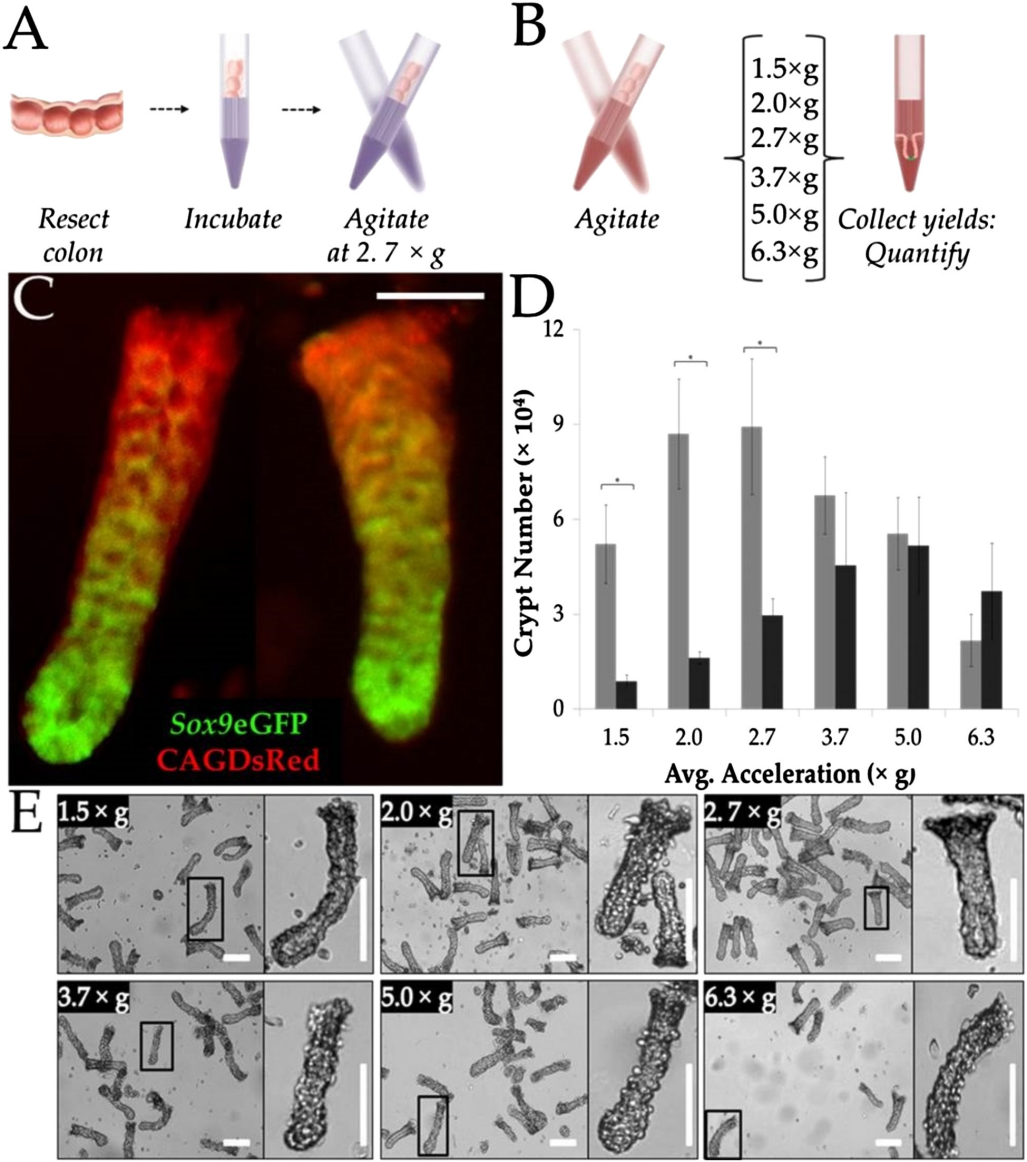
**Isolation of crypts from a mouse colon. (A)** Schematic of crypt isolation. The resected colon was incubated in chelating buffer, washed and then mechanically agitated. **(B)** Schematic of the strategy to identify the optimal acceleration intensity needed to retrieve crypts: the tissue was incubated in chelating buffer, rinsed and then sequentially agitated at different acceleration intensities. After each agitation, the crypt-rich supernatant was collected and assayed. **(C)** Fresh crypts isolated using an acceleration intensity of 1.5 × g displaying eGFP fluorescence, indicating *Sox9* expression (green). Scale bar = 40 μm. **(D)** Quantification of the number of intact and broken crypts at each of the six acceleration intensities tested. Grey bars indicate intact-crypt yield and black bars indicate broken-crypt yield. **(E)** Brightfield images of isolated crypts isolated at different accelerations intensities. Crypts isolated using an acceleration intensity of 1.5 × g display a visible lumen, indicating unperturbed crypt morphology. Scale bar = 150 μm.

In the initial step of crypt isolation, the colon is incubated in a buffer to chelate divalent cations and reduce disulfide bonds. Chelation of divalent cations reduces crypt-stromal adhesion by binding the calcium and magnesium ions required for receptor interactions between the basement membrane and stromal cells [3,20]. The chelation-buffer, incubation time was optimized by varying the time (30, 60, 90 min) in which the colon was placed in a standard buffer with EDTA and DTT, initially described by Booth *et al.* (Figure 1A) [20]. This buffer was chosen due to its past usage and reported high cell viability. All other isolation steps were held constant. Isolated crypts were assayed for the total yield of intact and broken crypts. The presence of intact crypts was used as an indicator of the extent of tissue trauma since these structures are easily fragmented when subjected to significant stress or harsh chemical conditions. Liberated crypts were considered to be intact if they were at least 150 μm in length. Utilization of a distal colon from the *Sox9*eGFP-CAGDsRed mouse model permitted facile evaluation of the viability of stem cell/progenitor (green plus red fluorescence) and differentiated lineages (red fluorescence) of the liberated crypts immediately after retrieval from the tissue [3]. The CAGDsRed mouse line, which ubiquitously expresses the red fluorescent protein DsRed, was bred with *Sox9*eGFP mice, which expresses eGFP under control of the *Sox9* promoter [3]. Previous work has demonstrated that the presence of the *Sox9* transcription factor is a distinguishing characteristic of colonic stem and progenitor cells.

Incubation of a single distal colon for 30 min in the EDTA-containing buffer resulted in a total yield of 139,000 ± 22,000 crypts of which 69.7% were intact. The 60-min incubation yielded 280,000 ± 28,000 crypts with 79.3% intact and 90-min incubation produced 360,000 ± 41,000 crypts with 65.9% intact. The 30-min incubation period provided the lowest yield of intact and total crypts, probably as a result of inadequate time for the chelating agents to be effective in disrupting submucosal adhesion. Although the 90-min incubation produced a higher overall yield, the 60-min incubation retrieved a higher percentage of intact crypts and resulted in more than double the number of intact crypts relative to that after a 30-min incubation. Therefore, a 60-min incubation was chosen for all subsequent experiments.

### Optimization of acceleration intensity required to release crypts

Following chelation of divalent ions and disruption of the adhesion between the epithelium and the basement membrane, mechanical agitation is use to remove crypts from the underlying tissue. Most protocols instruct “vigorous agitation of the tissue” to retrieve crypts, without quantification of the force or accelerative intensities involved [3,8,9,11,20]. To develop a reproducible protocol, varying average acceleration intensities were quantified for mechanically agitating the tissue in releasing crypts from the underlying stroma. Initially the average acceleration intensity achieved during agitation for 5 s was varied (1.5, 2.0, 2.7, 3.7, 5.0 and 6.3 × g) to optimize the agitation step (Additional file 1: Figure S1). To minimize the number of animals used, the colon was agitated for 5 s at the lowest acceleration intensity followed by settling of the tissue remnant and collection of the crypt-containing supernatant. Fresh isolation buffer was then added to the colon and the tissue was agitated at the next highest acceleration intensity after which the supernatant was again collected. This procedure was repeated until 6 crypt-containing supernatants were collected (Figure 1B). Each fraction was assayed for the total number of crypts, the number of intact crypts and crypt quality. Crypt quality was quantified by measuring the percentage of crypts that were both intact and retained an identifiable lumen. Identification of the lumen insured that the crypts possessed the basic morphology present *in vivo*. Utilization of the *Sox9*eGFP-CAGDsRed mouse model permitted verification of the quality, as only crypts possessing intact stem-cells possessed green fluorescence at the crypt base (Figure 1C). The total number of undamaged crypts with an identifiable lumen was greatest for acceleration intensities of 1.5, 2.0 and 2.7 × g (52,000 ± 15,000, 87,000 ± 17,000 and 90,000 ± 21,000 crypts, respectively) (Figure 1D). An ANOVA comparison revealed that the 1.5, 2.0 and 2.7 × g agitation acceleration intensities produced statistically different yields (p < 0.01) of intact versus broke crypts. The percentage of crypts with the appropriate morphology was optimal when the acceleration intensities were 1.5 × g (85.7%) and 2.0 × g (84.3%). At increased acceleration intensities (>3.7 × g), the lumens collapsed (Figure 1E). While progressively higher acceleration intensities liberated more crypts, the apparent quality of the crypts was also diminished as the acceleration intensities increased. For these measurements, the optimal compromise between crypt yield and quality was thus determined to be 1.5 and 2.0 × g. Since an untested combination of chelation-buffer incubation times and agitation conditions might have proved superior, a broad range of combinations of incubation times and acceleration intensities were assessed (Additional file 2: Table S1). Of the conditions tested, 60-min incubation in chelation buffer and an acceleration intensity of 1.5 × g yielded the greatest percentage of crypts with high-quality morphology. eGFP was expressed in 36 ± 4% of the crypt area, demonstrating that crypts isolated under these conditions possessed intact stem/proliferative cells (Additional file 3: Figure S2). Since the intended application of this work was the culture of viable crypts with formation of colonoids, these conditions were used for all subsequent experiments. When a higher yield of crypts is required without regard to quality, for example in gene expression studies, longer incubation times and greater acceleration intensities would generate significantly larger sample sizes and might be preferable.

### Optimization of matrigel concentration for colonoid culture

**Figure 2:**
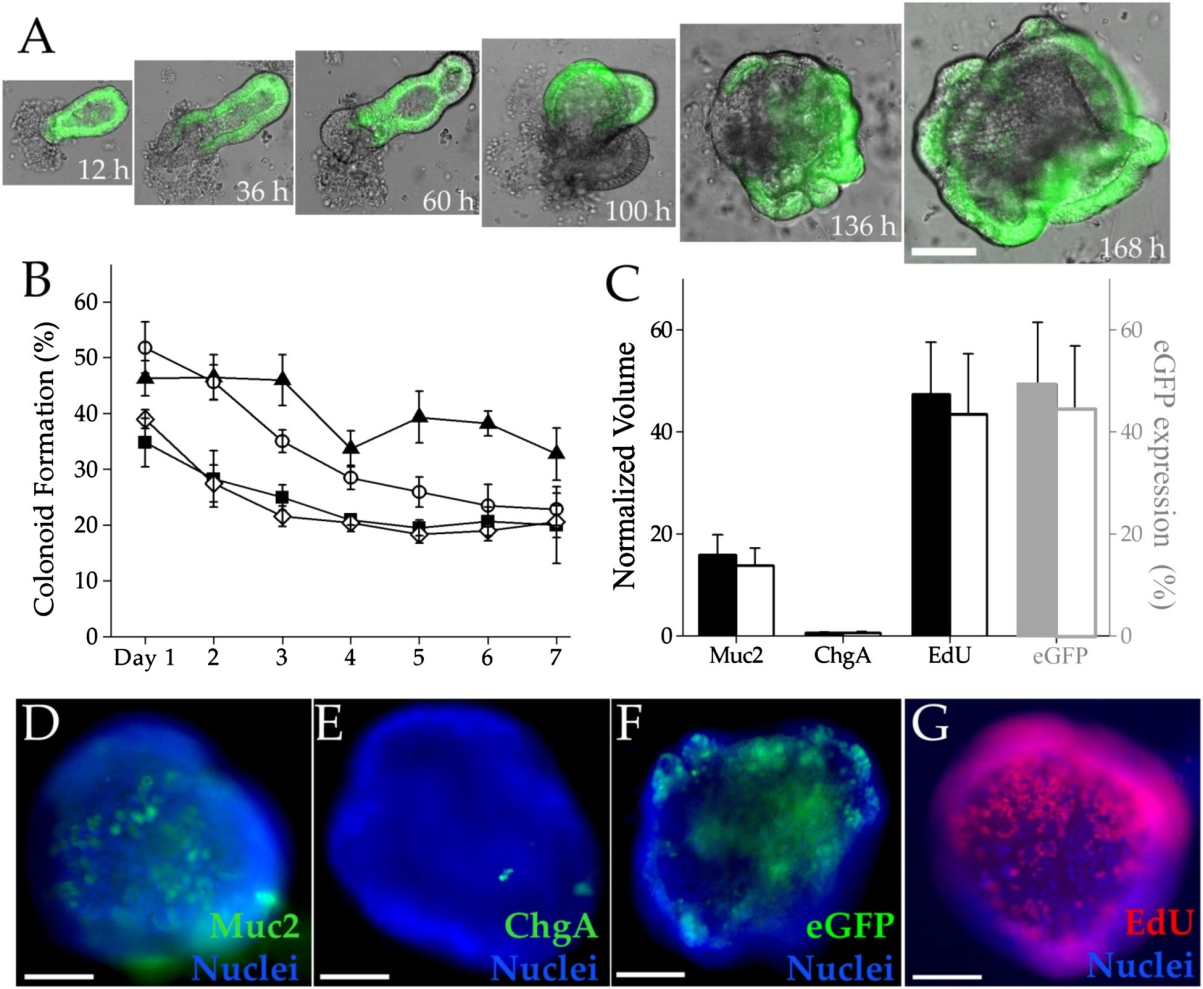
**Effect of Matrigel concentration on *in vitro *expansion of colonic crypts into 3-D colonoids. (A)** Serial overlaid brightfield and eGFP fluorescence images of the same colonoid over 1 week in culture. Scale bar = 50 μm. **(B)** Effect of Matrigel concentration on the percentage of crypts growing into colonoids over 1 week of culture. Squares, triangles, circles and diamonds represent 25%, 50%, 75% and 100% Matrigel, respectively. 50% Matrigel provides the optimum 3-D growth environment for the colonoids. **(C)** Quantification of the cell properties in the colonoids. Shown is the colonoid volume (left y axis, black) staining positive for Muc-2, ChgA or EdU divided by that positive for Hoechst 33342 when colonoids were cultured in 100% (filled bars) or 50% (open bars) Matrigel. The volume of the colonoid expressing eGFP relative to that expressing dsRed is shown on the right y-axis (grey) for colonoids cultured in 100% (filled bars) or 50% (open bars) Matrigel. **(D-E)** Colonoids were cultured for 1 week and then stained by immunohistochemistry for: **(D)** mucin-2 (goblet cell marker: green) and **(E)** chromogranin-A (enteroendocrine marker: green). **(F)** A crypt obtained from a *Sox9*eGFP-CAGDsRed mouse was cultured for 1 week and then imaged for eGFP fluorescence. **(G)** Fluorescence image of a colonoid (1 week culture) after an 8-hour EdU pulse (red). Hoescht 33442 was used as a nuclear stain (blue) in panels **C-G**. Scale bar = 75 μm.

Laminin-rich Matrigel is believed to provide the required matrix contacts for crypt cells mimicking that supplied by the underlying stroma *in vivo*[3,8,10]. Additionally, it is likely that Matrigel contains critical growth factors to maintain the crypt cells. In all past reports, crypts were cultured in 100% Matrigel, although it is unknown if this is the optimal concentration for colonoid growth. At 100%, Matrigel is extremely viscous, quick to gel and difficult to load into confined spaces such as those in microfabricated devices (*e.g.* microfluidic channels). For these reasons, four concentrations of Matrigel (25%, 50%, 75% and 100 vol% in complete culture medium (CCM) plus growth factors) were assessed for the ability to support colonoid formation. Crypts were isolated using the optimized protocol described above and were then plated on a microwell formed from native 1002-F such that the crypts remained suspended in Matrigel and not in contact with the 1002-F surface. The Matrigel-encapsulated crypts were imaged daily by brightfield and fluorescence microscopy (Figure 2A). The percentage of crypts forming colonoids was quantified as the number of budding crypts divided by the number of total crypts plated (n = 4 experiments for each Matrigel concentration with an average of 113 ± 32 crypts/experiment). Interestingly, 100% Matrigel was the least effective in yielding colonoid growth (18 ± 1%) after 7 days of culture in microwells (Figure 2B). 50% Matrigel supported the highest percentage of colonoid formation (33 ± 5%) followed by 75% and 25% Matrigel (23 ± 3% and 20 ± 7%, respectively) at day 7, as determined by colonoid morphology. To verify that crypts isolated under the optimal conditions and cultured in 50% Matrigel formed colonoids which possessed all of the differentiated cell lineages, immunostaining for the post-mitotic lineage markers Muc2 (mucus-producing goblet cells) and ChgA (hormone-secreting enteroendocrine cells) was performed (Figure 2D-E). For colonoids cultured in 50% Matrigel, eGFP was expressed in 49 ± 14% of the colonoid volume compared to 49 ± 12% in 100% Matrigel, suggesting similar numbers of stem/progenitor cells at one week under both conditions (Figure 2F). The colonoid volume positive for Muc2 or ChgA was 14 ± 3 and 0.6 ± 0.2 times the volume staining positive for Hoechst 33342 when cultured in 50% Matrigel for 7 days. In the presence of 100% Matrigel for 7 days, Muc2^+^ or ChgA^+^ regions occupied 16 ± 4 and 0.5 ± 0.2 times more volume than that of Hoechst 33342 suggesting that the density of these differentiated cell types was similar for the two conditions. Colonoids in 50% and 100% Matrigel possessed EdU^+^ positive regions (43 ± 12 and 47 ± 10 times greater in volume than that positive for Hoechst 33342) suggesting that comparable numbers of cells were actively synthesizing DNA when cultured in the two different Matrigel concentrations (Figure 2G) [22]. Thus colonoids cultured in 50% Matrigel were nearly identical to that in 100% Matrigel with respect to these measured cell properties.

Since the use of 50% Matrigel was superior to the other concentrations at forming colonoids and was also able to support both stem/proliferative and differentiated cells, 50% Matrigel was employed in all subsequent experiments. Given the high cost of Matrigel, reduction in the concentration to 50% will substantially reduce future experimental costs. The mechanistic impact of Matrigel concentration on the cells is unknown; however, the optimal concentration identified in this study suggest that 50% Matrigel may provide the optimal stiffness, the proper concentrations of growth and differentiating factors, and/or the appropriate density of extracellular matrix contacts to maximize colonoid cell growth.

### Assessment of crypt interaction with microfabricated substrates

Surface biochemical properties are known to modulate the growth and differentiation of stem cells [23-26]. Thus, the property of solid surfaces in contact with the crypts is likely to impact the efficiency of colonoid formation and potentially the fate of the crypt cells. Along with the 3-D colonoids, it was noticed that the intestinal crypt cells also formed 2-D monolayers when in contact with the well substrate. Conditions promoting monolayer formation from primary cells for this monolayer have not been well described [9,11,27,28]. For this reason, five commonly used transparent, microfabrication substrates were assessed for their impact on cell growth and phenotype: glass, polystyrene, PDMS, and the epoxy photoresists SU-8 and 1002-F. Glass and polystyrene have long been the gold standard for cell culture, and devices can be microfabricated by a variety of methods from all of these materials. PDMS is the most popular material for prototyping microdevices and it can be readily microfabricated by soft lithography [13,29,30]. The epoxy-based, transparent, negative SU-8 photoresist is used in building high-aspect ratio microstructures by standard photolithography [31]. 1002-F photoresist is closely related to SU-8 in molecular structure, and prior work has demonstrated that 1002-F is biocompatible, supporting cell attachment and growth, and exhibits significantly lower autofluorescence than SU-8 [31-33]. Non-transparent or opaque substrates (*e.g.* silicon) were not assessed here due to their incompatibility with many light microscopy methods.

**Figure 3:**
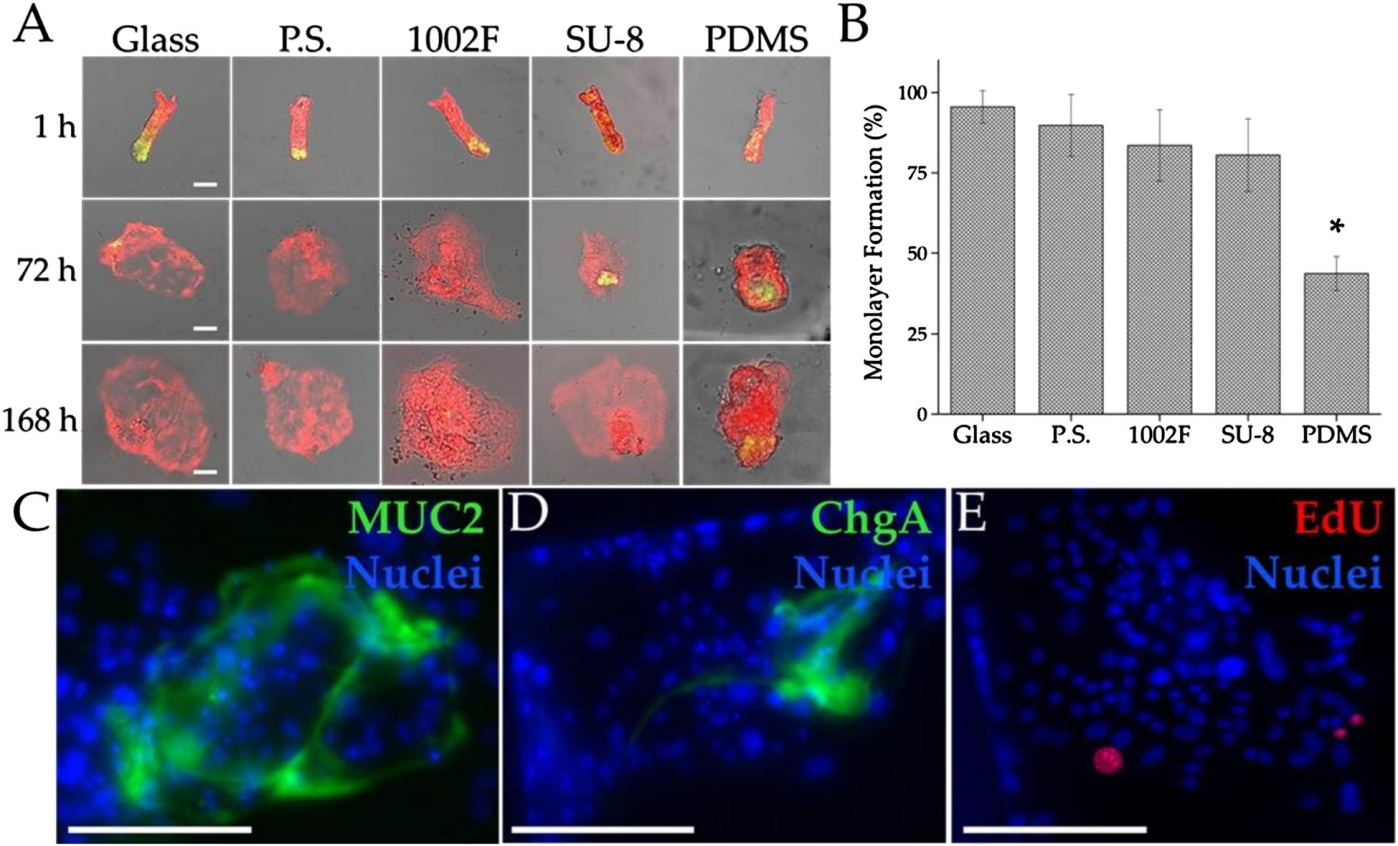
**Crypt-substrate interaction. (A)** Representative time-lapse images of monolayer formation on the experimental substrates from crypts isolated from a *Sox9*eGFP-CAGDsRed mouse. eGFP and DsRed fluorescence was overlaid on brightfield microscopy images. Upon adherence to glass, oxidized polystyrene and epoxy photoresist, crypt-cells rapidly differentiate. **(B)** Quantification of the percentage of crypts forming a monolayer when crypts were cultured on the microfabrication substrates over 1 week. **(C, D)** Whole-mount immunohistochemical staining of a monolayer after 1 week in culture. Fluorescence images are shown for: mucin-2 (green, **C**) and chromogranin-A (green, **D**). **(E)** Fluorescence image after an 8-hour EdU pulse (red). Hoescht 33442 was used as a nuclear stain (blue) in panels **C-E**. Scale bars = 50 μm.

Crypts were cultured in contact with the microfabrication substrates and monolayer expansion efficiency was calculated by dividing the number of crypts that successfully expanded into monolayers by the total number of crypts plated. 95.5 ± 2.5% of crypts plated on glass developed into monolayers, the highest average percentage of any of the experimental materials. After one week, 46.3 ± 3.4% of crypts cultured on native PDMS substrates developed into monolayers, the lowest percentage of the experimental materials (p-value of 5.67 × 10^−6^) (Figure 3A). However, monolayer-formation percentage for crypts on glass was not statistically different than that on polystyrene, 1002-F and SU-8 (p-values of 0.33, 0.10 and 0.052, respectively) (Figure 3B). Immunohistochemical staining for the goblet-cell and enteroendocrine lineages demonstrated the presence of differentiated cells throughout the monolayers on the PDMS surfaces (Figure 3C-D).

The monolayers forming on the various surveyed substrates possessed very little eGFP fluorescence, suggesting little to no *Sox9* expression. Monolayers on 1002-F and PDMS substrates possessed the most eGFP expression after a week of culture (covering 1.3 ± 0.8% and 3.7 ± 3.0% of the monolayer surface area, respectively). To determine whether cells in the monolayers were proliferating but without eGFP expression, an EdU-based cellular proliferation assay was performed on the cells grown for 7 days on PDMS substrates. EdU^+^ cells were infrequent in the monolayers suggesting that most of the cells within the monolayer were not actively proliferating (compare Figure 3E to 2G). These data suggested that crypt-cells rapidly differentiated upon adherence to glass, oxidized polystyrene and epoxy photoresists. All of these materials are hydrophilic displaying charged oxygen groups on their surface. It may be necessary to avoid cell contact with these surfaces to maintain stem/progenitor cells. PDMS was an exception likely because the intrinsic hydrophobic properties of PDMS [30] discouraged surface attachment by cells. PDMS may be attractive for microfabricated devices constructed to house stem/progenitor cells. Further studies will be required to understand how the surface property of a substrate modulates the fate of crypt cells.While monolayer formation was critically dependent on the surface properties of the culture vessel, monolayer dependence on the overlaid Matrigel concentration was less pronounced as long as a concentration threshold of 50% Matrigel was utilized (Additional file 4: Figure S3).

## Conclusions

The current work established a reproducible, standardized isolation protocol for isolating intact murine colonic crypts with high proliferative capacity. In a step-wise fashion, the incubation duration of the tissue in chelating buffer and mechanical acceleration intensities required for crypt release were optimized to retrieve the maximal number of high quality crypts. The concentration of Matrigel, a costly reagent used for *in vitro* expansion of intestinal stem cells, was optimized to maximize the development of colonoids from the isolated crypts while minimizing reagent use. Crypts were isolated from a genetically engineered *Sox9*eGFP-CAGDsRed reporter mouse, enabling fluorescent measurements to be used as functional readouts of stem-cell proliferation and differentiation. The microwell provided an efficient platform for facile screening and quantification of colonoid formation while further reducing the amounts of expensive reagents including such as Wnt-3a, EGF, Noggin, and R-spondin1. Immunohistochemical staining demonstrated the presence of the differentiated intestinal cellular lineages (goblet and enteroendocrine) in these colonoids. The type of growth (2-D monolayer *vs*. 3-D colonoid) was dependent on the culture substrate properties. Crypts plated on PDMS substrates demonstrated the highest percentage of 3-D colonoid formation and most stem cells, while crypts plated on glass, polystyrene, 1002-F and SU-8 surfaces produced the highest percentages of 2-D monolayer formation with few identifiable stem cells. By standardizing the isolation process and optimizing the matrix concentrations on different surfaces, reproducible crypt isolation and robust culture protocols were established to facilitate the use of colonoid-based assays by the intestinal stem-cell community. Common microfabricated substrates were surveyed to identify substrates that are compatible with maintenance of stem and differentiated cells. This research provides a clear isolation and culture protocol for colonic crypts supporting future development of intestinal studies and ‘organ-on-chip’ endeavors.

## Methods

### Materials

N2 and B27 supplements, GlutaMAX, Advanced DMEM/F12 base media, 5-ethynyl-2′-deoxyuridine (EdU) kit and α-goat-Alexa Fluor 488 were purchased from Invitrogen (Carlsbad, CA). Y27632 Rock inhibitor, HEPES buffer, N-acetylcysteine (NAC), ethylenediaminetetraacetic acid (EDTA, 0.5 M, pH 8.0), bis-Benzimide (Hoescht 33342), and α–rabbit-Cy3 were obtained from Sigma-Aldrich (St. Louis, MO). Cell-culture-grade bovine serum albumin (BSA), dithiothreitol (DTT) and a pulse vortex-mixer were purchased from Thermo-Fisher (Fairlawn, NJ). Recombinant mouse Wnt-3a, recombinant human R-Spondin1 and recombinant mouse EGF were acquired from R&D Systems (Minneapolis, MN). Growth-factor reduced Matrigel was obtained from BD Biosciences (Bedford, MA). Recombinant mouse Noggin was purchased from Peprotech (Rocky Hill, NJ). Sylgard 184 silicone elastomer kit was procured from Dow Corning (Midland, MI). EPON epoxy resin 1002-F (fusion solids) was purchased from Miller Stephenson Chemical Co. (Sylmar, CA). Primary antibodies α-mucin2 and α-chromogranin A were purchased from Santa Cruz Biotechnology (Santa Cruz, CA).

### Transgenic mouse model and isolation of colonic crypts

The *Sox9*eGFP-CAGDsRed mouse model on a CD-1 background was used for experimental analysis. The CAGDsRed mouse line ubiquitously expresses the red fluorescent protein DsRed under the control of a chicken beta-actin promoter (CAG). The DsRed-expressing mice were bred with *Sox9*eGFP mice, which possessed the *Sox9* promoter controlling eGFP (enhanced green fluorescent protein) expression on a modified bacterial artificial chromosome (BAC) [3]. Previous work demonstrated that *Sox9* is expressed in the stem and progenitor cells of the colon so that the *Sox9*eGFP mouse possesses eGFP expression in the stem/proliferative cell compartment at the crypt base. For stem/progenitor cell quantification within fresh crypts, monolayers and colonoids, eGFP fluorescence was used as a measure of *Sox9* expression After resecting the distal colon from a 6–9 week-old mouse, the colon was cut longitudinally, flushed of its contents and washed with chilled rinse buffer (5.6 mM Na_2_HPO_4_, 8.0 mM KH_2_PO_4_, 96.2 mM NaCl, 1.6 mM KCl, 43.4 mM sucrose, 54.9 mM D-sorbitol, pH 7) [20]. The distal colon was then incubated in isolation buffer (rinse buffer + 2.0 mM EDTA + 0.5 mM DTT), for 30, 60 or 90 min at 22ºC as indicated in the text. The tissue was washed by transferring to 3 separate vials containing chilled rinse buffer. The sample was then agitated at 2.7 × g for 5 seconds using a pulsing vortex mixer, unless stated otherwise in the text. The crypts were inspected by brightfield microscopy for the presence of a defined lumen. The overall yield was determined by adding a 250 μL crypt-suspension to a 12-well plate and using a 4× objective to count the number of crypts per field of view. This number was then used to calculate the number of crypts in the total volume of crypt-suspension.

### Calculation of acceleration intensity

The pulsing vortex mixer (Fisher Scientific, Catalog # 02-215-375) produced acceleration profiles in the x-, y-, and z-directions. The acceleration of 6 different settings on the vortex mixer (800–1800 rpm) was measured over time using a 3-D accelerometer (Gulf Coast Data Concepts, Catalog# X16-1C). The magnitude of the x-, y-, and z-direction acceleration vectors was calculated from these measurements. The average magnitude over time was then used as the average accelerated intensity.

### Culture of colonic crypts for matrigel optimization

A microwell fabricated from thick 1002-F photoresist was used for facile tracking of the colonoids to optimize the Matrigel concentration. To assess the effect of Matrigel on colonoid formation, Matrigel was diluted in complete culture medium (CCM: advanced DMEM-F12 with N2 supplement, B27 supplement, 1× GlutaMAX, 10 μM HEPES buffer, 1 μg/mL penicillin, 1 μg/mL streptomycin, 3.2 mg/mL Y27632 and 163.2 mg/mL NAC) at 4°C to yield 25, 50, 75 and 100 vol% concentrations. The microwells were sterilized with ethanol, washed × 3 in rinse buffer, and placed at 4°C before plating the crypts. A 400-μL suspension of crypts was added to each microwell (5000 crypts/mL) and the crypts were allowed to settle into the wells for 2 min. The supernatant was then carefully removed and ice-cold Matrigel (400 μL) was overlaid. The Matrigel was supplemented with the following growth factor concentrations: 5 ng/mL Wnt-3a, 50 ng/mL EGF, 100 ng/mL Noggin and 1 μg/mL R-spondin1 [3]. Matrigel was polymerized for 15 min at 37°C. After polymerization, 1.6 mL of complete culture medium was overlaid onto the Matrigel. Growth factors were replenished by direct addition to the medium every 2 days and the medium was changed every 4 days. R-spondin1 was used at 1 μg/mL for the initial plating and 500 ng/mL for the duration of the culture. Y27632 and NAC were only included in the CCM at the time of initial plating and were removed from subsequent culture media.

### Culture of crypts on microfabrication substrates

Five different common microfabrication substrates were tested for the culture of crypts on their surface: PDMS, polystyrene (tissue-culture treated), glass, and the photoresists SU-8 and 1002-F. Round glass coverslips (#1, diameter = 25 mm) were spin-coated with PDMS, SU-8 or 1002-F, baked and cured, sterilized with ethanol, and placed in a 6-well plate. Before culturing, the substrates were coated with 50% Matrigel at 4°C for 8 h. A 200 μL crypt suspension (5000 crypts/mL) in Matrigel (50% in CCM unless otherwise stated) was added to each of the 6 wells. The plate was then placed at 4°C for 10 min to ensure that the crypts traveled through the liquid gel and settled onto the experimental substrate. Subsequently, the gel was polymerized at 37°C for 15 min. After polymerization, the crypts were overlaid with CCM. Growth factor and media exchange was performed as described above.

### EdU analysis and immunostaining

Crypts and colonoids from wild-type mice were used for EdU analysis [22] and immunostaining. 5-ethynyl-2′-deoxyuridine (EdU) nucleoside was used to assess proliferation as per manufacturer’s instructions: EdU (10 μM) was added to the culture medium and allowed to become incorporated into the cells for 4 h. The culture medium was then removed, and the entire culture was fixed using 3.7% paraformaldehyde in phosphate-buffered saline (PBS) for 20 min at room temperature. Cells in the fixed colonoids were permeabilized using 0.5% Triton X-100 in PBS for 20 min, followed by washing × 3 with PBS containing 3% BSA. Each quadrant of the microwell was then incubated with 250 μL of click-it reaction cocktail (containing the Alexa Fluor-555 azide) for 30 min at room temperature, followed by rinsing × 3 with PBS. The samples were stored in PBS at 4°C until visualization by fluorescence microscopy. For immunostaining, colonoids were fixed, rinsed with PBS and permeabilized using 0.3% Triton X-100 in PBS for 20 min. Following rinsing × 3 with PBS containing 100 mM glycine, the colonoids were incubated in immunofluorescence (IF) wash (0.2% Triton X-100, 0.1% BSA, 0.05% Tween-20, 7.7 mM NaN_3_ in PBS and 5% normal goat serum) for 90 min to block nonspecific binding. Primary-antibodies (α-chromogranin A and α-mucin2) were applied in IF wash (1:100) for 12 h at 4°C. Secondary antibodies (α-rabbit-Cy3 and α-goat-Alexa Fluor 488) were applied in IF wash (1:500) for 45 min. All nuclei were stained with bis-benzimide (10 μg/mL in PBS) using a 30 min incubation [3].

### Image analysis of monolayers and freshly isolated crypts

Epifluorescence images were captured on a Nikon Eclipse TE2000-U microscope fitted with a Photometrics CoolSNAP HQ2 digital camera. Objective lenses used were 10×, 20× and 40× with numerical apertures of 0.30, 0.55 and 1.40, respectively. Prior to quantification, image acquisition and preprocessing of raw images was necessary to reduce background noise. This was done using a custom script implemented in MATLAB (MathWorks; Natick, MA) for each fluorescence image acquired. Background was first reduced using a top-hat filter followed by application of a median filter to smooth the images and further reduce noise. The images were then thresholded and ‘holes’ were filled to create a binary image which was used to define the image area with measurable fluorescence. The total number of pixels in this masked area was then summed for each image. Quantification for the regions of stem/proliferative cell area within monolayers and freshly isolated crypts were assessed by dividing the number of eGFP^+^ pixels by the total number of DsRed^+^ pixels in the image. All data points represent the average ± standard deviation of at least four separate experiments. Statistical analysis was conducted by one-way ANOVA pairwise tests. A p-value of < 0.05 was considered statistically significant.

### Image analysis for the colonoids

Confocal images were captured on a Zeiss CLSM 710 Spectral Confocal Laser Scanning Microscope, using objective lenses of either 20× or 40× magnifications (numerical apertures of 0.80 and 0.95, respectively). Preprocessing of the raw images, thresholding and masking was performed for each confocal slice as described in the previous section. eGFP was quantified relative to DsRed in each slice as described in the prior section and then averaged over all slices possessing colonoids to yield the percentage colonoid volume positive for eGFP. To quantify the Muc2- and ChgA-expression or EdU-staining regions in each image slice, the number of pixels positive for these markers was divided by the number of pixels positive for Hoechst 33342. The average ratio for every slice in a sample was then calculated to yield the average volume of sample positive for Muc2, ChgA, or EdU relative to that positive for Hoechst 33342.

## Competing interests

The authors declare they have no competing interests or conflicts.

## Authors’ contributions

All authors were involved in the design of the experiments and writing the manuscript. AA performed the experiments. This work is in partial fulfillment of the Doctoral Degree requirements of AA. All authors read and approved final manuscript.

## Supplementary Material

Additional file 1: Figure S1 Accelerometer measurements of the acceleration vector magnitudes applied to the colonic tissue. Click here for file

Additional file 2: Table S1 At different average acceleration intensities **(A)** the total number of whole crypts isolated and the **(B)** percentage of crypts isolated with intact morphology. Click here for file

Additional file 3: Figure S2 Effect of increased accelerated agitation intensity on eGFP expression. Click here for file

Additional file 4: Figure S3 Effect of Matrigel concentration on monolayer expansion. Data collected on native PDMS surfaces. Squares, circles, triangles, and x’s represent 25%, 50%, 75% and 100% Matrigel, respectively. Click here for file
