# Supplementary figures and images for: Optimization of 3-D organotypic primary colonic cultures for organ-on-chip applications
Source: J Biol Eng. 2014 Apr 1;8:9. doi: 10.1186/1754-1611-8-9 (PMC4022271; doi:10.1186/1754-1611-8-9)

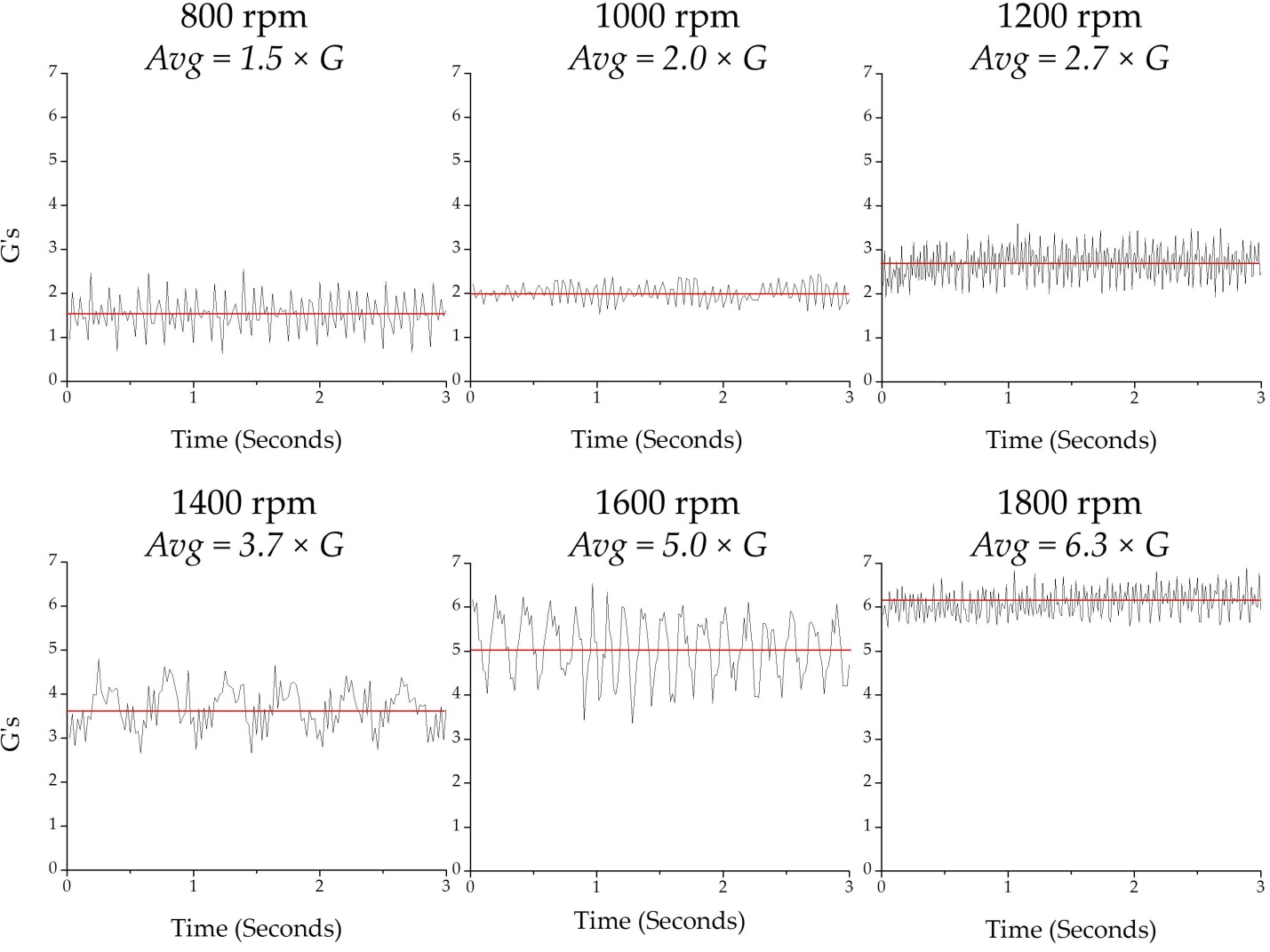

Supplement: Additional file 1: Figure S1 — Accelerometer measurements of the acceleration vector magnitudes applied to the colonic tissue. [file 1754-1611-8-9-S1.doc]

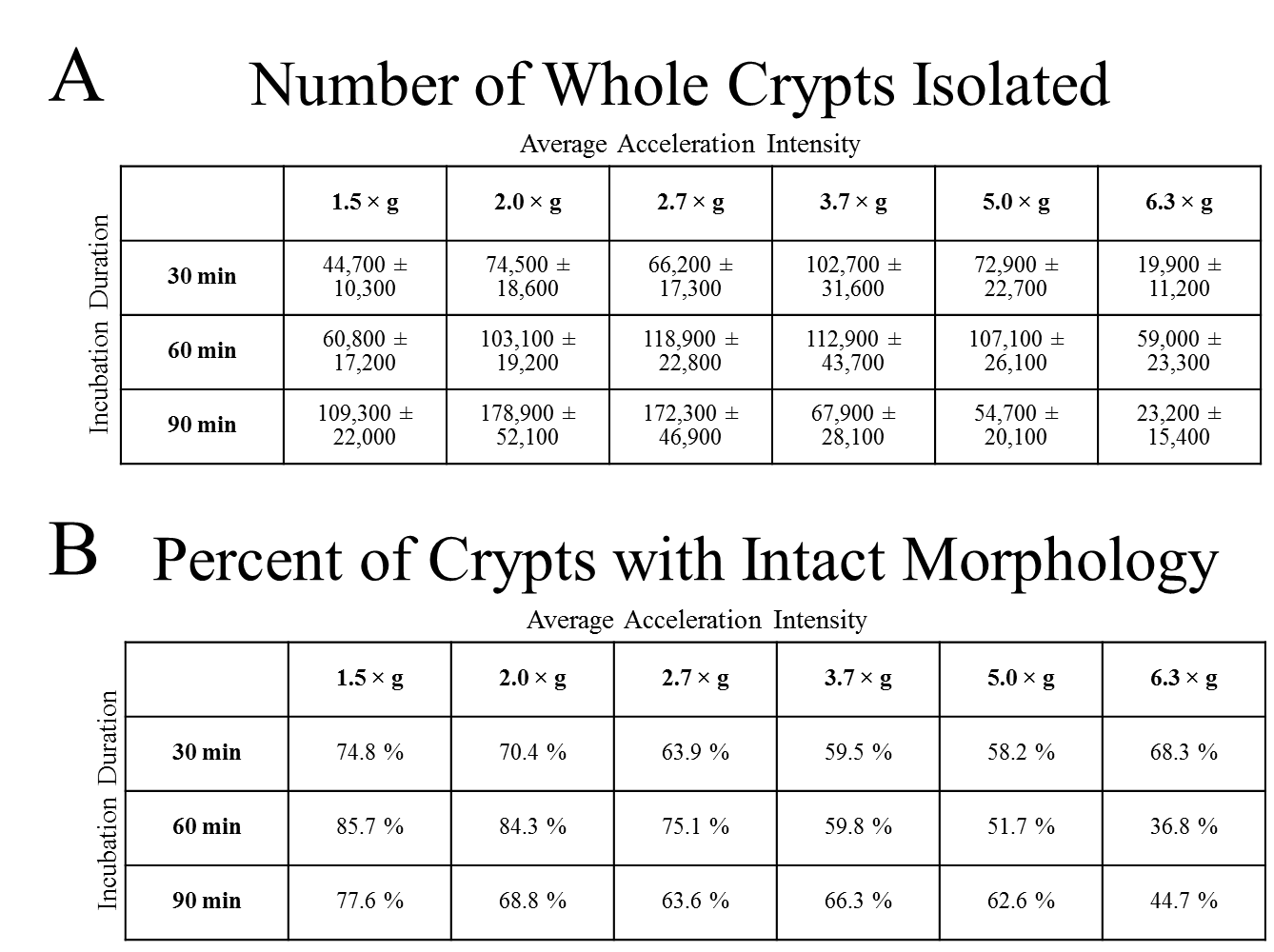

Supplement: Additional file 2: Table S1 — At different average acceleration intensities (A) the total number of whole crypts isolated and the (B) percentage of crypts isolated with intact morphology. [file 1754-1611-8-9-S2.doc]

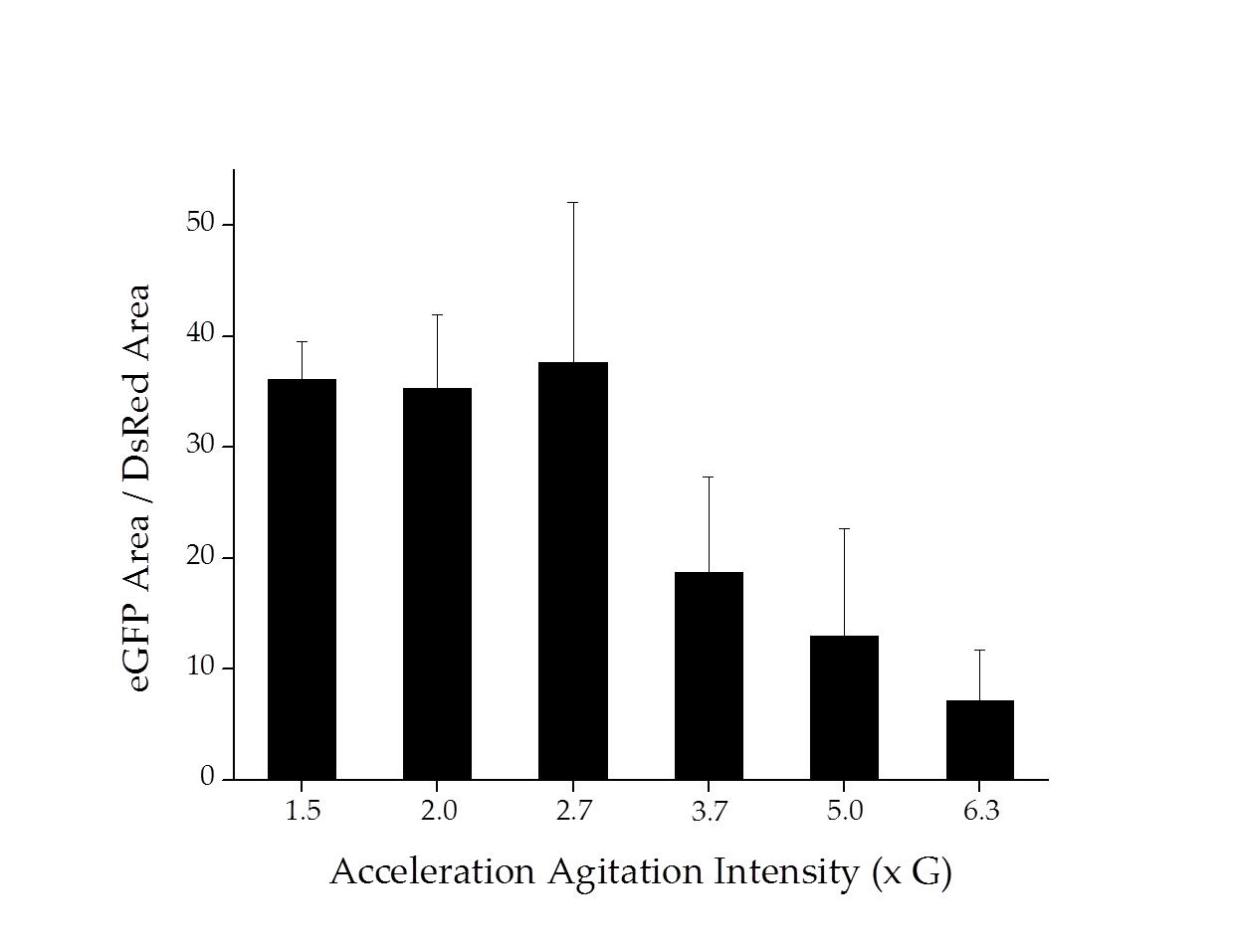

Supplement: Additional file 3: Figure S2 — Effect of increased accelerated agitation intensity on eGFP expression. [file 1754-1611-8-9-S3.doc]

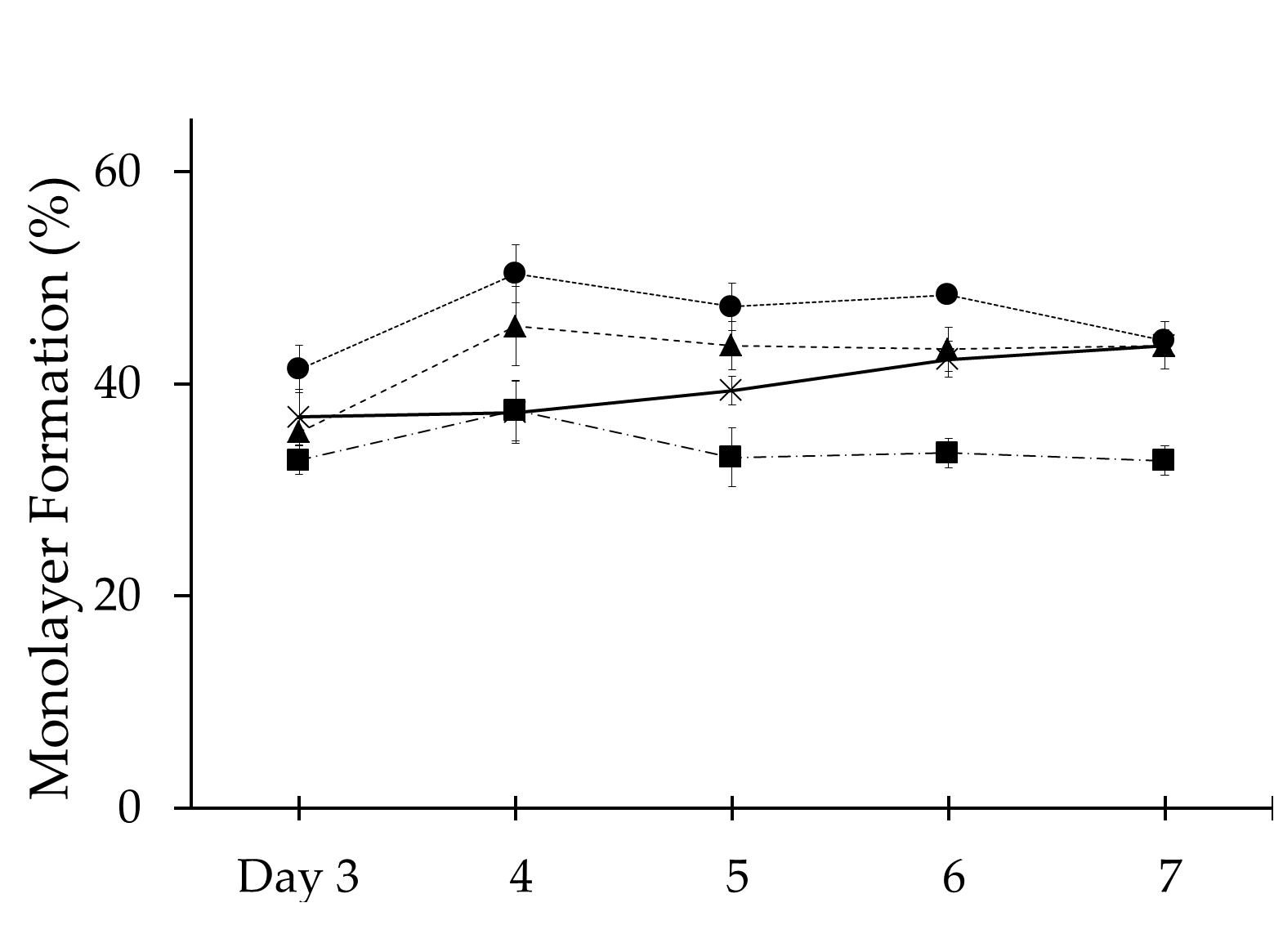

Supplement: Additional file 4: Figure S3 — Effect of Matrigel concentration on monolayer expansion. Data collected on native PDMS surfaces. Squares, circles, triangles, and x’s represent 25%, 50%, 75% and 100% Matrigel, respectively. [file 1754-1611-8-9-S4.doc]
